# Prevalence and factors influencing influenza vaccination among health care workers in China: a systematic review and meta-analysis

**DOI:** 10.3389/fpubh.2026.1831275

**Published:** 2026-09-21

**Authors:** Lina Xiong, Chunfang Cheng, Jie Yun

**Affiliations:** 1 College of Nursing, Chengdu University of Traditional Chinese Medicine, Chengdu, China; 2 Dazhou Central Hospital, Dazhou, Sichuan, China; 3 Hospital of Chengdu University of Traditional Chinese Medicine, Chengdu, China

**Keywords:** healthcare workers, influencing factors, influenza vaccination, meta-analysis, systematic review, vaccination coverage

## Abstract

**Aims:**

To systematically evaluate the influenza vaccination rate among health care workers in China and identify influencing factors.

**Design:**

Systematic review and meta-analysis of cross-sectional studies.

**Data Sources:**

CNKI, Wanfang, VIP, Web of Science, PubMed, Cochrane Library, Scopus, and Embase, searched from database inception to October 4, 2025.

**Review Methods:**

Two reviewers independently screened studies, extracted data, and assessed study quality using the Agency for Healthcare Research and Quality (AHRQ) tool. Random-effects models were used to pool vaccination rates and odds ratios. Heterogeneity was assessed using I^2^ statistics, with supplementary subgroup analysis, meta-regression, and sensitivity analysis. Publication bias was assessed via funnel plot and Egger's test.

**Results:**

Forty-nine studies involving 111,544 participants were included. The pooled influenza vaccination rate was 27% (95% CI: 21–34%). Factors positively associated with vaccination included: age 40–49 years, female gender, monthly income > 3,000 RMB, professional title, prior vaccination history, institutional encouragement or requirement, free vaccination policies, awareness of optimal vaccination timing, and vaccination willingness.

**Conclusion:**

Influenza vaccination coverage among Chinese health care workers remains inadequate. Structural interventions-such as free vaccination, institutional encouragement, and on-site services-combined with tailored strategies targeting younger, male, low-income, and vaccine-naïve individuals are recommended. Given the observational nature of the included studies, the potential role of mandatory policies should be interpreted cautiously, pilot implementation in high-risk settings is suggested before broader adoption.

**Impact:**

This study provides an evidence base for formulating targeted influenza vaccination strategies for health care workers in China, which is crucial for reducing nosocomial infection risk and aligning with global public health goals.

**Patient or Public Contribution:**

No patient or public contribution.

**Systematic review registration:**

PROSPERO registration CRD: 42024553631.

## Introduction

Influenza vaccination among health care workers plays an important role in the prevention and control of global infectious diseases and the stable operation of the medical service system. The World Health Organization has set the influenza vaccination rate of health care workers as 75% (1). Based on their elevated occupational exposure risk, role in nosocomial transmission to vulnerable patients, and the critical need to maintain healthcare system function during influenza epidemics. Health care workers have also been listed as a priority vaccination group in the “Technical Guidelines for seasonal influenza Vaccination in China (2025–2026)” by the Chinese Center for Disease Control and Prevention (2). It aims to reduce the risk of nosocomial infection and provide protection for susceptible patients. The current vaccination rate in China is still not up to the standard, and there are significant differences in different regions and medical institutions. According to the surveillance data of the Chinese Center for Disease Control and Prevention, the influenza vaccination rate of health care workers has been lower than 30% for a long time, and about 15%-30% of health care workers are infected in the annual influenza season, which seriously affects the safety of medical services (3). Vaccination rates are affected by individual, hospital and system levels. For example, some areas include vaccination as an evaluation index of nosocomial infection and provide free vaccination, while other areas rely on self-paid vaccination. This imbalance exposes a large number of medical workers to the risk of infection and easily misses the opportunity to prevent influenza and its complications (4). Low influenza vaccination uptake among HCWs is not unique to China but represents a global challenge, particularly in low- and middle-income countries (LMICs). A recent systematic review and meta-analysis reported a global HCW influenza vaccination rate of 41.7%, with rates of 46.9% in low-income countries and 35.6% in high-income countries (5). However, country-specific studies reveal substantial variations and persistent barriers. In South Africa, vaccine unavailability and hesitancy were identified as the main reasons for low uptake among HCWs caring for the older adults (6). In Thailand, influenza vaccine hesitancy was highly prevalent among HCWs in a northeastern province (7). In Tunisia, a cross-sectional survey during the 2018–2019 season confirmed low vaccination rates and underscored the need for tailored education programs (8). In India, multiple barriers, contribute to persistently low coverage (9). These findings from other LMICs, which share similar health system constraints and cultural contexts with China, highlight the importance of understanding country-specific determinants to inform effective interventions. The economic value of HCW influenza vaccination has also been demonstrated. A systematic review and meta-analysis of cost-effectiveness studies found that influenza vaccination is cost-effective for HCWs in high-income countries compared with no vaccination or current practice (10). However, evidence from low and middle-income countries remains limited.

Most of the current studies are limited to a single cross-sectional survey, with insufficient sample size and representation, and heterogeneity in the identification of factors affecting vaccination in different studies (11). Against this background, this study conducted a systematic review and meta-analysis of the influenza vaccination rate and its influencing factors among health care workers in China. This study provides an evidence-based basis for formulating accurate influenza vaccination strategies for health care workers, which is helpful for influenza prevention and reducing the risk of nosocomial infection. This study is consistent with the global goals for influenza prevention and treatment.

## Methods

### Protocol and ethics review

This study was conducted in accordance with the PRISMA (Preferred Reporting Items for Systematic Reviews and Meta-Analyses) statement (12) and registered on PROSPERO, CRD: 42024553631.

### Literature search strategy

Cross-sectional studies on The influencing factors of influenza vaccination among health care workers in China were searched by computer in China National Knowledge Infrastructure (CNKI), VIP, Wanfang Data, Web of Science, PubMed, The Cochrane Library, Embase and other databases, and references and gray literature were searched retrospectively. The search time limit was from the establishment of the database to October 4, 2025. To supplement electronic database searches, we hand-searched reference lists of included studies and relevant reviews, and screened the first 10 pages of Google Scholar results using the same search terms. To avoid duplicate inclusion of the same participant populations, we systematically compared potentially related publications by author names, study periods, sample sizes, and study settings. If overlap was confirmed, we retained only the study with the largest sample size or most complete data and excluded the others. Detailed search strategies are shown in Supplemental material S1.

### Inclusion criteria

(1) Study population: healthcare workers (HCWs) in Chinese hospital or community settings;
(2) Outcome measures: influenza vaccination data;
(3) Study results: complete data reporting odds ratios (ORs) and 95% confidence intervals (CIs) for influencing factors;
(4) Study design: cross-sectional studies;
(5) Original articles published in Chinese or English.

### Exclusion criteria

The criteria outlined below were employed to exclude studies from this meta-analysis:

(1) articles without full text;
(2) reviews, systematic reviews, and conference abstracts;
(3) duplicate publications, studies reusing data, or studies with unavailable data.

### Literature screening and data extraction

Following deduplication in EndNote X9, two researchers trained in evidence-based methodology independently conducted literature screening and quality assessment. Discrepancies were resolved by a third researcher, and data extraction was performed after consensus. Extracted data comprised: first author, publication year, region, study population, survey period, sampling type, sample size, and influencing factors. After deduplication using EndNote X9 (via the “Find Duplicates” function), two reviewers independently screened the remaining records. Candidate influencing factors were prespecified based on a preliminary literature review and theoretical frameworks. For meta-analysis, we included only factors reported in≥3 studies, with consistent definitions. To handle cross-study variability, we standardized categorical definitions using a uniform re-coding scheme (e.g., age into < 30, 30~39, 40~49, ≥50; income dichotomized at 3,000 RMB; professional title into none/primary/medium/advanced; hospital policies into three harmonized categories). All coding decisions were made independently by two reviewers.

### Evaluation of quality in included studies

Cross-sectional studies were evaluated using the Agency for Healthcare Research and Quality (AHRQ) recommended criteria (13), consisting of 11 items scored as 1 for “yes” and 0 for “no,” “unclear,” or “not applicable.” Overall study quality was categorized as low (score ≤ 3), moderate (score 4–7), or high (score ≥8). Discrepancies in quality assessment were resolved by a third researcher.

### Statistical analysis

Stata 17.0 software was used for all statistical analyses, the test level α was set at 0.05. The pooled influenza vaccination rate and the odds ratios (ORs) for influencing factors, along with their 95% confidence intervals (CIs), were calculated as effect estimates.Cochran's Q test was used to analyze the combined effect size and confidence interval, and I^2^ test was used to test the heterogeneity. When *P* ≥ 0.10 and *I*^2^ ≤ 50%, the fixed effect model was used to analyze the homogeneity of the study. When *P* < 0.10 and *I*^2^ > 50%, the random-effects model was used to analyze the heterogeneity between studies. To explore potential sources of heterogeneity, pre-specified subgroup analyses were conducted based on the local economic development level, sample source, survey time period, and sampling method. Univariate meta-regression was performed to quantify the association between these covariates and the estimated vaccination rates. The robustness of the pooled estimates was evaluated through sensitivity analysis by sequentially excluding individual studies. Publication bias was assessed visually using a funnel plot and quantitatively confirmed by Egger's linear regression test. A two-sided *P* < 0.05 was considered statistically significant.

## Results

### Study screening results

A total of 726 literatures were obtained in the initial inspection, 391 literatures were left after importing EndNote X9 to remove the duplicate, 76 literatures were left after reading the title and abstract, 27 articles were excluded after further reading the full text, and 49 literatures were finally included after strict evaluation. The literature screening process is shown in Figure 1.

**Figure 1:**
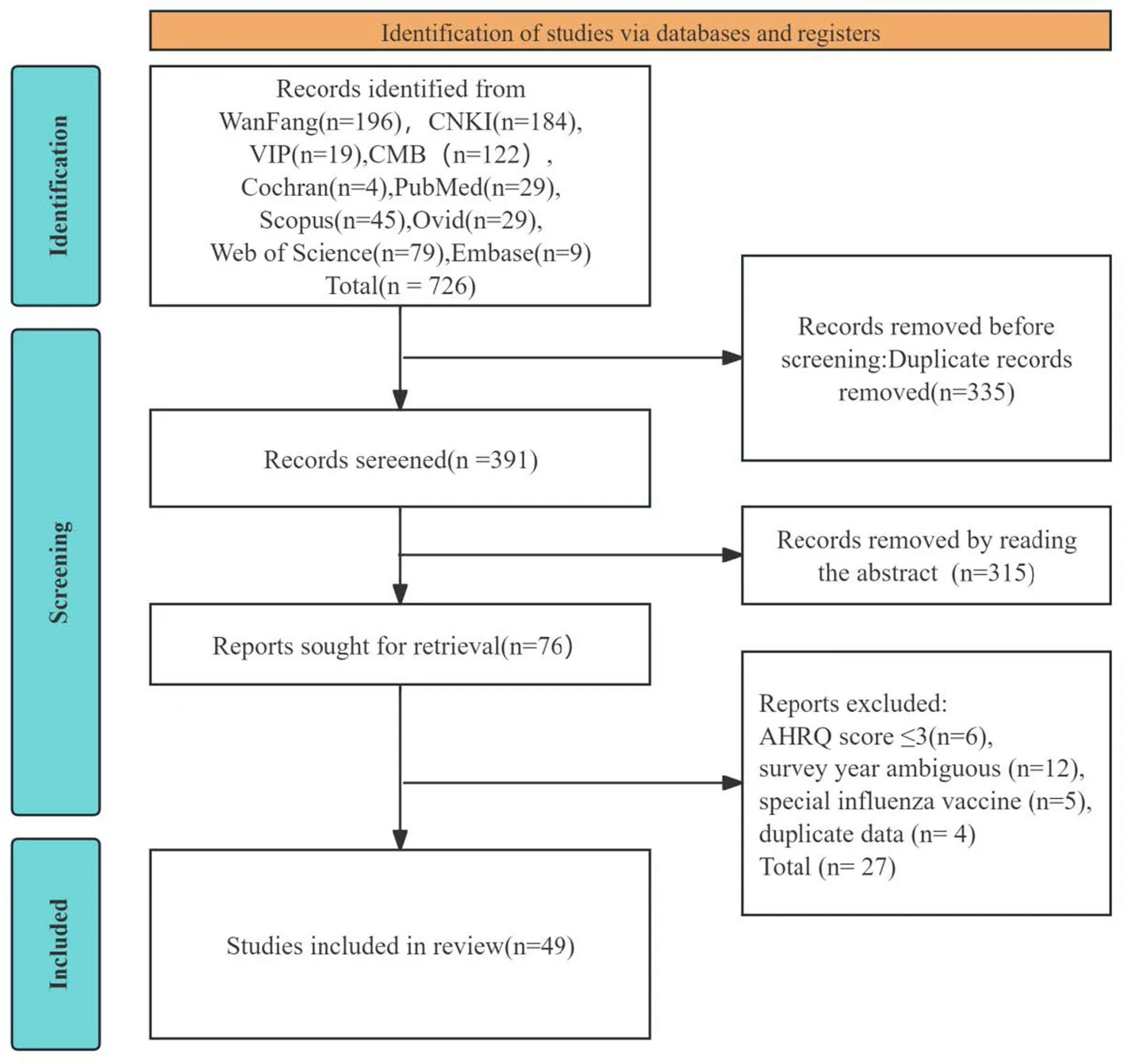
PRISMA flow diagram of the studies selection process.

### Basic characteristics of included studies and quality assessment results

Forty nine studies reported influenza vaccination status among healthcare workers, totaling 111,544 participants. 40 studies identified influencing factors, including age, gender, professional title, influenza vaccination policies, and influenza vaccination knowledge. The basic characteristics and methodological quality assessment results of the included studies are presented in Table 1.

**Table 1:** Basic characteristics and quality evaluation results of the included literature.

Study ID	Areas	Sample source	Survey time	Sampling type	AHRQ	Influencing factors	Vaccination rate (n/N)
Bu et al. (16)	Shandong	Hospital	2012	Random	7	①②③④	6.5% (98/1,521)
Bu et al. (17)	Shandong	Hospital	2013	Random	7	①⑤⑥⑦	4.8% (63/1,301)
Song et al. (18)	Liaoning	Hospital	2015	Random	8	①⑤⑧	9.12% (95/1,042)
Ma et al. (19)	Shandong	Hospital	2012	Random	9	①⑤⑥	6.45%(98/1,520)
Kan et al. (20)	Shanghai	Hospital	2011	Convenience	8	①⑨⑩	8.8% (79/895)
Yang et al. (21)	Qinghai	Hospital	2016	Random	9	⑩⑪⑫⑬	5.51% (107/1,941)
Ma et al. (22)	Qinghai	Hospital	2017	Random	8	⑩⑪⑭	6.80% (226/3 260)
Xu et al. (23)	Qinghai	Hospital	2018	Random	7	⑧⑮⑯⑰	17.22% (705/4,094)
Liu et al. (24)	Beijing	Hospital	2018	Convenience	9	⑧⑪⑫⑭⑰	31.20% (424/1 359)
Sheng et al. (25)	Heilong jiang	Hospital	2017	Convenience	8	①⑧⑮⑯⑱	4.01% (31/773)
Yang et al. (26)	Shandong	Hospital	2018	Random	8	⑤⑥⑨⑱	6.64% (42/633)
Zhang et al. (27)	Multicenter	Hospital	2019	Random	7	①⑥⑬⑯⑱⑲⑳㉑	11.57% (472/4,078)
Luo et al. (28)	Chongqing	Hospital	2018	Random	9	①⑧⑨	16.78% (237/1,412)
Yi et al. (29)	Multicenter	Hospital	2019	Random	8	①⑧⑬⑲⑳㉑	67% (2,927/4,366)
Jiang et al. (30)	Sichuan	Hospital	2018	Random	8	⑤⑲⑳㉒	7.69% (168/2,186)
Kong et al. (31)	Multicenter	Community	2019	Random	9	①⑱㉓㉔	24.97% (2 241/8 975)
Li et al. (32)	Multicenter	Hospital	2020	Random	7	①⑧⑰⑱⑲⑳㉔㉕	59.5% (2,294/3,856)
Lv et al. (33)	Shanghai	Community	2018	Convenience	7	⑥⑧⑨⑰⑲	14.57% (216/1 483)
Zhang et al. (34)	Shanghai	Hospital	2018	Random	8	⑥⑧⑫⑲	13.89% (131/943)
Ma et al. (35)	Multicenter	Hospital	2021	Random	9	⑧⑰⑲⑳㉓	35.4% (600/1,697)
Shi et al. (36)	Ningxia	Hospital, Community	2020	Convenience	8	⑧⑪⑬⑲㉔㉕㉓	39.6% (868/2,192)
Yang et al. (37)	Chongqing	Community	2020	Random	8	⑤⑧	46.2% (528/1,142)
Fan et al. (38)	Qinghai	Hospital	2019	Random	7	⑰⑳	15.58% (1,037/6,654)
Hu et al. (39)	Zhejiang	Hospital	2020	Random	7	①⑤⑧⑲	35.28% (121/343)
Jiang et al. (40)	Guangzhou	Hospital	2019	Random	8	①⑳㉔	23.97%(713/2,974)
Lei et al. (41)	Guizhou	Hospital	2020	Random	7	⑩⑪⑬	22.60%(419/1,854)
Yan et al. (42)	Multicenter	Hospital	2019	Convenience	8	①⑤⑧⑨㉔㉗	46.09% (614/1 332)
Zhang et al. (43)	Gansu	Hospital	2019	Random	8	①⑨㉓	32.90% (255/775)
Sun et al. (44)	Shandong	Community	2021	Random	8	⑤⑰⑱㉗	65.2% (1 232/1 889)
Yang et al. (45)	Guangxi	Hospital	2020	Random	8	①⑧⑱⑲㉔㉘	27.13% (599/2,208)
Liu et al. (46)	Hainan	Hospital	2024	Convenience	8	⑤⑬⑯⑰	62.91% (2,280/3,624)
Kang et al. (47)	Sichuan	Hospital	2022	Convenience	7	①⑥⑱⑲㉔	56.90% (1,846/3,244)
Shi et al. (48)	Zhejiang	Hospital	2023	Random	7	⑧⑯㉕	22.99% (183/796)
Li et al. (49)	Guangdong	Hospital	2023	Convenience	7	⑤⑬⑱⑲㉔	66.01% (4,483/6,791)
Tu et al. (50)	Jiangxi	Hospital	2023	Random	7	⑲⑳	19.37% (153/790)
Zhang et al. (51)	Beijing	Hospital	2024	Random	8	⑧⑱⑲	43.26% (77/178)
Shen et al. (52)	Zhejiang	Hospital	2024	Convenience	8	⑲	1.36% (30/2,205)
Zheng et al. (53)	Zhejiang	Hospital	2022	Random	8	①⑤⑬	22.60% (49/217)
Liu et al. (54)	Shandong	Hospital	2021	Random	7	①⑱⑲⑳㉑㉔㉕	67.50% (8,761/12,985)
Gao et al. (55)	Beijing	Hospital	2013	Convenience	7	㉑㉖㉙	13.93% (51/366)
Huang et al. (56)	Shandong	Community	2012	Random	7	①③⑭㉖	12.88% (30/233)
Wang et al. (57)	Beijing	Hospital	2012	Random	7	⑨⑳㉙	30.1% (171/569)
Yang et al. (58)	Guangdong	Hospital	2013	Convenience	8	⑨㉖㉙	43.69% (284/650)
Yu et al. (59)	Multicenter	Hospital	2017	Random	8	⑪⑳㉑㉖	6% (257/4,153)
Jiao et al. (60)	Chongqing	Hospital	2018	Convenience	8	⑨⑬⑱㉙	19.69% (189/960)
Liu et al. (61)	Beijing	Hospital	2018	Convenience	9	⑤⑬㉔	22.74% (68/299)
Wang et al. (62)	Shanxi	Hospital	2017	Convenience	7	⑯⑰⑱	3.1% (16/510)
Fan et al. (63)	Shandong	Community	2020	Random	8	①⑤⑯㉔	87.13% (670/769)
Wu et al. (64)	Shandong	Hospital	2018	Convenience	7	①⑨㉘㉚	11.8 % (413/3,507)

① Hospital level, ② awareness of vaccination time, ③ chronic disease, ④ perceived safety of influenza vaccine, ⑤ history of influenza vaccination, ⑥ intention to receive influenza vaccination, ⑦ concern about contracting influenza, ⑧ department, ⑨ knowledge of influenza vaccination, ⑩ awareness of annual influenza vaccination, ⑪ perceived effectiveness of influenza vaccine, ⑫ awareness of priority recommendation for healthcare workers, ⑬ professional title, ⑭ awareness that influenza vaccination is the most effective means of preventing influenza, ⑮ gender, ⑯ years of work experience, ⑰ free influenza vaccination provided by hospital, ⑱ age, ⑲ occupation, ⑳ hospital attitude toward influenza vaccination, ㉑ free influenza vaccination policy, ㉒ smoking status, ㉓ monthly income, ㉔ education level, ㉕ concern about vaccine side effects, ㉖ geographic location, ㉗ health status, ㉘ inconvenience of influenza vaccination, ㉙ colleague recommendation.

### Meta-analysis results of the influenza vaccination rates among health care workers

A meta-analysis of the influenza vaccination rate of 49 articles showed that the overall influenza vaccination rate of HCWs was 27% (95% CI = 21%−34%), as shown in Figure 2. Heterogeneity test showed *I*^2^ = 99.91%, *P* < 0.001. To explore the sources of heterogeneity and identify the key factors affecting vaccination rates, subgroup analysis and meta-regression were performed.

**Figure 2:**
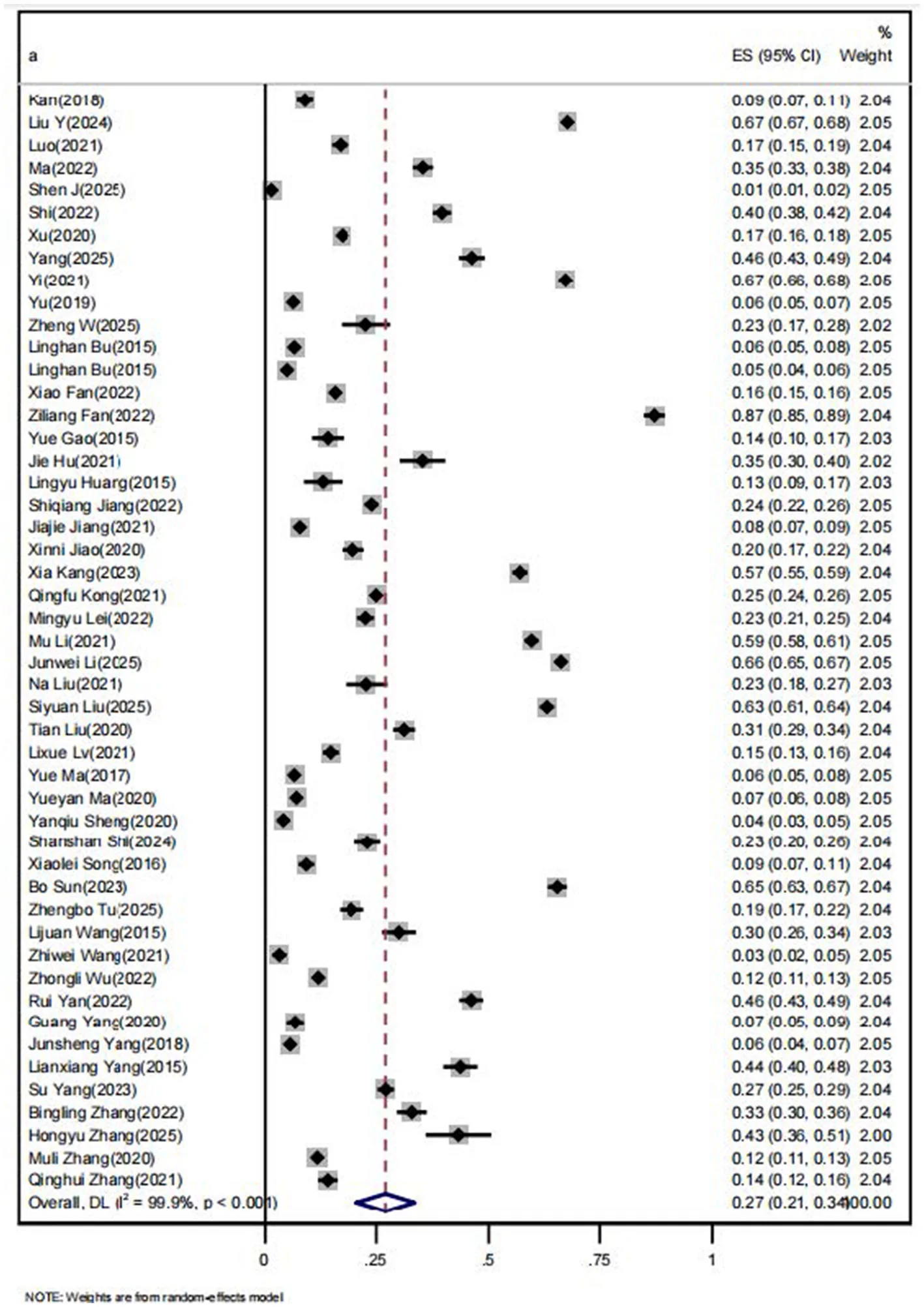
Meta-analysis results of influenza vaccination rates.

### Subgroup analysis and meta-regression results of influenza vaccination coverage

The information of all included literatures was extracted, and subgroup analysis was conducted by region, publication year, survey population, sample source, sampling method, and AHRQ score. Provinces were classified into three categories, economically developed, moderately developed, and underdeveloped, based on the National Bureau of Statistics of China's official four-region classification (eastern, central, western, and northeastern). According to this standard, the eastern region (Beijing, Tianjin, Hebei, Shanghai, Jiangsu, Zhejiang, Fujian, Shandong, Guangdong, and Hainan) was designated as economically developed; the central region (Shanxi, Anhui, Jiangxi, Henan, Hubei, and Hunan) as moderately developed; and the combined western (Inner Mongolia, Guangxi, Chongqing, Sichuan, Guizhou, Yunnan, Tibet, Shaanxi, Gansu, Qinghai, Ningxia, and Xinjiang) and northeastern (Liaoning, Jilin, and Heilongjiang) regions as underdeveloped. Studies were categorized into three intervals: 2011–2015, 2016–2020, and 2021–2024. These cutoffs were chosen based on key policy and epidemiological milestones in China's influenza vaccination landscape. For monthly income, we dichotomized at 3,000 RMB based on (i) the most frequently reported cutoff in the original studies included in this meta-analysis, and (ii) the national income classification framework, in which 3,000 RMB approximates the lower threshold of middle-income groups in China. This threshold has been widely adopted in Chinese health behavior research to differentiate income levels (14). For vaccination payment policy, we categorized studies into self-paid vs. free vaccination. This classification follows the distinction established in the Chinese CDC technical guidelines and prior studies on HCW influenza vaccination.

The results are detailed in Table 2. The vaccination rate was highest in the central south region (44.02%, 95% CI = 28.95%−60.29%), and lowest in the northeast region (6.17%, 95% CI = 3.44%−10.81%). The vaccination rate in eastern and northwest China was also at a low level. The vaccination rate showed a significant upward trend over time: the pooled vaccination rates in 2011–2015, 2016–2020 and 2021–2024 were 12.13%, 20.52%, and 36.36%, respectively, indicating a significant increase in influenza vaccination coverage among health care workers in recent years. The vaccination rate from the community sample was higher than that from the hospital sample. Studies using random sampling reported higher vaccination rates than studies without random sampling. No significant differences in vaccination rates were observed among studies with different AHRQ scores.

**Table 2:** Subgroup analysis and meta-regression of influenza vaccination rates among HCWS in China.

Subgroup	Number	Rate of vaccination	*95%CI*	*I^2^*	β	*P*
Local economic level						
Economically developed	21	30.20%	11.70%, 43.20%	99.90%	—	
Moderately developed	10	25.40%	11.10%, 39.70%	99.90%	−0.59	0.562
Underdeveloped	10	17.40%	11.40%, 23.40%	99.50%	−1.56	0.128
Sample source						
Community	6	40.20%	18.43%, 66.65%	99.80%	—	
Hospital	42	19.17%	14.11%, 25.52%	99.70%	1.01	0.983
Survey time						
2011–2015	9	12.13%	7.30%, 19.47%	99.00%	—	
2016–2020	29	20.52%	14.35%, 28.45%	99.70%	1.65	0.237
2021–2024	11	36.36%	19.89%, 56.81%	99.60%	3.4	0.006
Sampling type						
Random sampling	33	26.70%	19.00%, 34.40%	99.90%	—	
Convenience sampling	16	27.90%	14.80%, 41.00%	99.90%	0.19	0.854

Meta-regression further quantified the effects of the above factors on the variability of vaccination rate: year of survey was a significant predictor (*P* < 0.001). The reported coverage rate was significantly higher during 2021–2024 than during 2011–2015 (β = 3.40, *P* = 0.006), confirming the annual increase trend. Region, sample source, sample type and AHRQ score did not show statistical significance in the meta-regression (all *P* > 0.05), indicating that these factors had no significant moderating effect on the combined effect size.

### Results of meta-analysis of factors associated with influenza vaccination rate

Factors potentially associated with influenza vaccination rates, including age, sex, professional title, vaccination policy, and vaccination knowledge, were analyzed. The results are presented in Table 3.

**Table 3:** Meta-analysis of factors associated with influenza vaccination rates.

Influencing factors	Number	Layers	Combined result		Heterogeneity		
			*OR*	*95%CI*	*P*	*I^2^*	*P*
Age	6	< 30	1.00				
		30–39	1.11	0.82, 1.51	0.494	75.00%	0.001
		40–49	1.36	1.02, 1.80	0.034	61.00%	0.025
		≥50	1.22	0.84, 1.76	0.296	76.80%	0.001
Gender	6	Female	1.43	1.03, 1.97	0.032	88.70%	< 0.001
Monthly income	3	>3,000	1.37	1.06, 1.78	0.016	73.40%	0.023
Professional title	6	No	1.00				
		Primary	1.21	1.00, 1.46	0.047	0.00%	0.884
		Medium	1.23	1.00, 1.50	0.045	0.00%	0.684
		Advanced	1.35	1.08, 1.69	0.008	36.10%	0.116
Occupations	10	Doctor	1.00				
		Nurse	1.04	0.87, 1.25	0.626	63.00%	0.004
		Medical technician	1.18	0.93, 1.49	0.166	62.20%	0.010
History of Vaccination	11	Yes	2.73	1.18, 6.34	0.019	98.20%	< 0.001
Hospital attitudes to vaccination	5	Don't ask and don't encourage	1.00				
		Not required but encouraged	1.72	1.51, 1.96	< 0.001	1.70%	0.397
		Requirement for vaccination	3.06	2.63, 3.56	< 0.001	0.00%	0.716
Free influenza vaccination	6	Yes	3.03	2.08, 4.40	< 0.001	91.30%	< 0.001
Free vaccination policy	3	Yes	3.20	1.52, 6.73	0.002	97.60%	< 0.001
Awareness of vaccination time	4	Yes	2.81	1.46, 5.41	0.002	88.20%	< 0.001
awareness of priority recommendation for healthcare workers	3	Yes	1.36	0.61, 3.05	0.451	89.10%	< 0.001
Awareness of annual influenza vaccination	3	Yes	2.31	0.33, 16.33	0.400	93.60%	< 0.001
Vaccination intention	5	Willing	6.66	4.03, 11.02	< 0.001	72.00%	0.007
Perceived effectiveness of influenza vaccine	3	Yes	0.93	0.34, 2.56	0.895	88.70%	< 0.001

### Socio-demographic and occupational factors

Compared with healthcare workers aged < 30 years, those aged 40–49 years had significantly higher vaccination likelihood (OR = 1.36, 95% CI = 1.02–1.80, *P* = 0.034). No statistical differences were observed for the 30-39 years group (OR = 1.11, 95% CI = 0.82–1.51) and ≥50 years group (OR = 1.22, 95% CI = 0.84–1.76). Female healthcare workers had 43% higher vaccination likelihood than their male counterparts (OR = 1.43, 95% CI = 1.03–1.97, *P* = 0.032). Monthly income > 3,000 RMB was associated with higher influenza vaccination likelihood (OR = 1.37, 95% CI = 1.06–1.78, *P* = 0.016).

Compared with those without professional title, healthcare workers with junior (OR = 1.21, 95% CI = 1.00–1.46, *P* = 0.047), intermediate (OR = 1.23, 95% CI = 1.00–1.50, *P* = 0.045), and senior titles (OR = 1.35, 95% CI = 1.08–1.69, *P* = 0.008) showed significantly higher vaccination likelihood. No statistical differences were found between physicians and nurses (OR = 1.04, 95% CI = 0.87–1.25) or medical technicians (OR = 1.18, 95% CI = 0.93–1.49).

### Vaccination history and policy factors

History of influenza vaccination was a strongly associated with vaccination uptake. Healthcare workers with previous vaccination were 2.73 times more likely to receive vaccination again compared with those without prior history (OR = 2.73, 95% CI = 1.18–6.34, *P* = 0.019). Hospital attitude toward vaccination demonstrated significant impact. Compared with “neither requiring nor encouraging,” “not requiring but encouraging” increased vaccination likelihood by 72% (OR = 1.72, 95% CI = 1.51–1.96, *P* < 0.001), while “requiring vaccination” increased it by 2.06-fold (OR = 3.06, 95% CI = 2.63–3.56, *P* < 0.001). Free vaccination was another strong facilitating factor. Compared with self-paid vaccination, free vaccination increased vaccination likelihood by 2.03-fold (OR = 3.03, 95% CI = 2.08–4.40, *P* < 0.001). The pooled effect size for free vaccination policy was similar (OR = 3.20, 95% CI = 1.52–6.73, *P* = 0.002).

### Knowledge and belief factors

Knowledge of vaccination schedule was significantly associated with higher vaccination likelihood (OR = 2.81, 95% CI = 1.46–5.41, *P* = 0.002). However, knowledge of “healthcare workers as a priority recommendation group” (OR = 1.36, 95% CI = 0.61–3.05) or “need for annual vaccination” (OR = 2.31, 95% CI = 0.33–16.33) showed no statistical association with vaccination behavior. Intention to receive vaccination was the strongest predictor of vaccination behavior. Healthcare workers who expressed willingness to vaccinate were 6.66 times more likely to actually receive vaccination than those unwilling (OR = 6.66, 95% CI = 4.03–11.02, *P* < 0.001). Belief that “vaccine is an effective measure for influenza prevention” showed no significant association with vaccination behavior (OR = 0.93, 95% CI = 0.34–2.56, *P* = 0.895).

### Sensitivity analysis and publication bias

Sensitivity analysis by sequentially removing individual studies showed minimal influence of single studies on the overall pooled effect size, indicating robust and reliable results. According to our findings, none of the studies had a significant effect on the pooled prevalence of influenza vaccination rates among HCWS in China (Figure 3).

**Figure 3:**
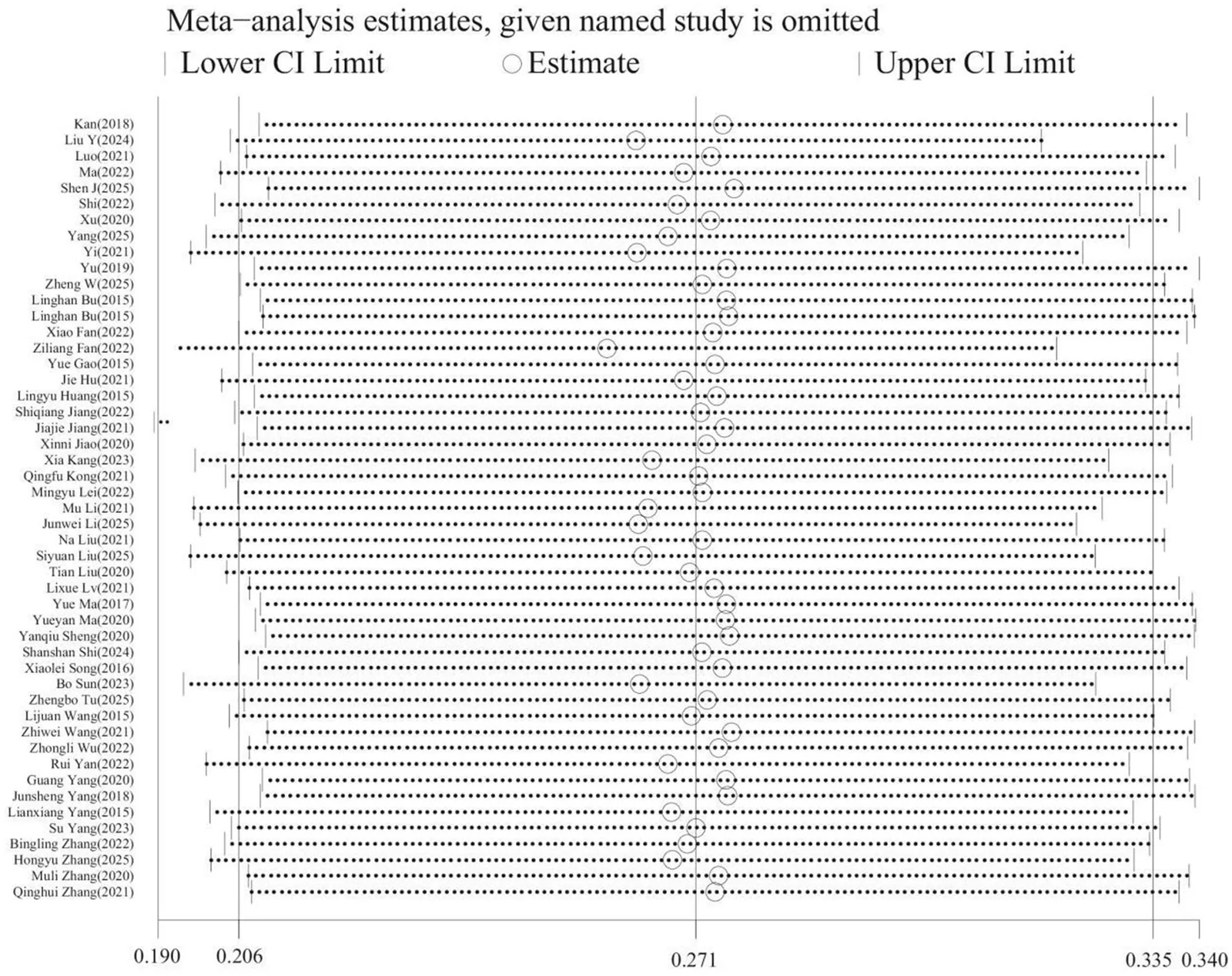
Results of sensitivity analysis.

A funnel plot was used to visually evaluate the potential publication bias of the influenza vaccination coverage estimates. The images show an asymmetric distribution of effect estimates around pooled means. Relatively few studies were observed in the lower right region of the figure, which typically corresponds to smaller studies with non-significant or low effect estimates. This visual asymmetry suggests a potential underrepresentation of such studies in the published literature, suggesting a possible publication bias (Figure 4).

**Figure 4:**
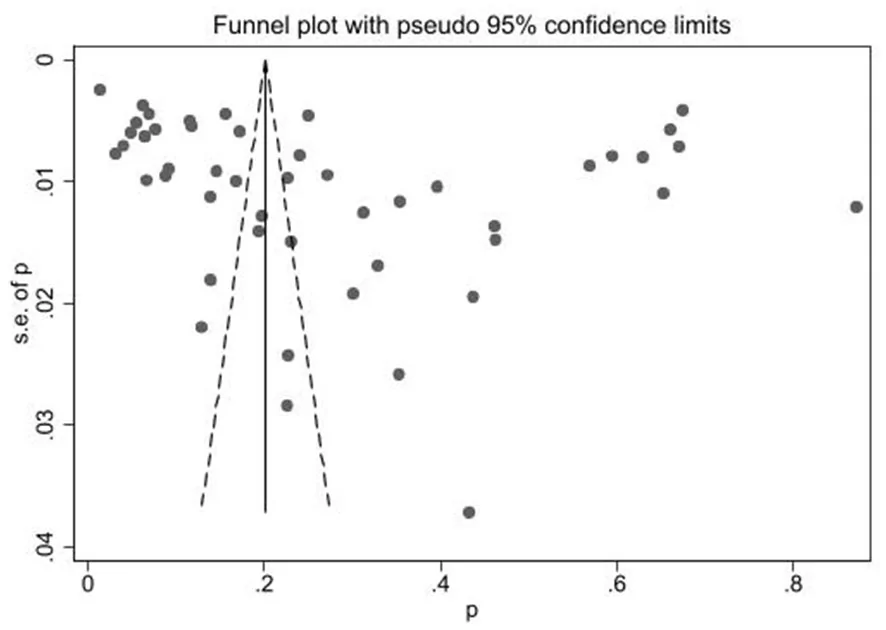
Funnel plot.

Egger's asymmetry test showed publication bias (intercept = 2.34, 95% CI: 0.85–3.82, *p* = 0.008), suggesting that studies with small sample sizes tended to report higher willingness to receive vaccination (Figure 5). This asymmetry raises the concern that the pooled estimate of 27% may be overestimated, as studies with null or negative findings could be systematically underrepresented in the published literature. However, since publication bias tests cannot definitively quantify its impact on the pooled estimate, this finding should be interpreted with caution rather than as a definitive correction.

**Figure 5:**
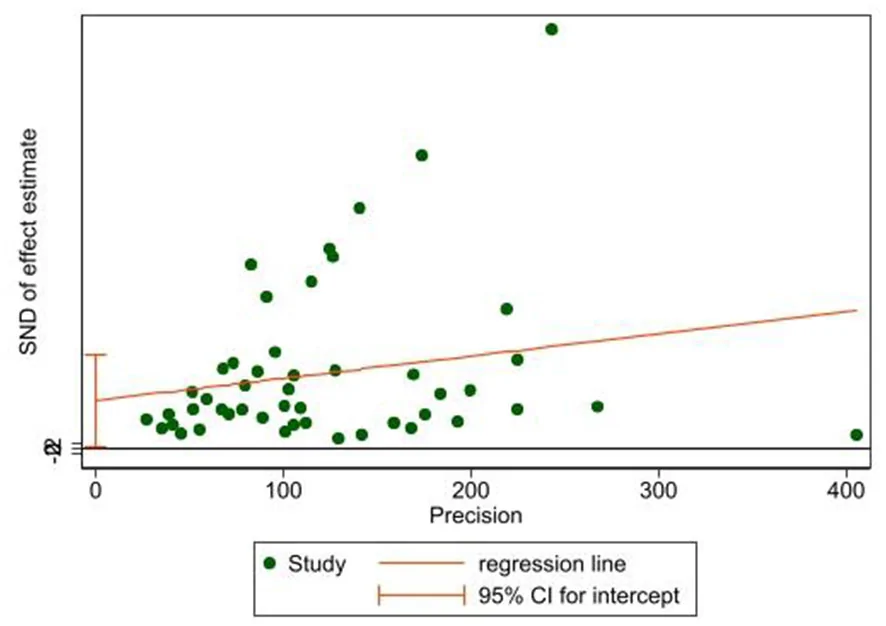
Egger regression analysis.

The presence of bias suggests that the 27% vaccination rate may overestimate the actual vaccination rate among healthcare workers in our country. Studies reporting higher vaccination rates or larger sample sizes appear more likely to be published. However, this interpretation remains speculative, as publication bias tests cannot establish the exact magnitude or direction of bias. Importantly, the robustness of our primary findings is supported by sensitivity analyses, which indicated that no single study exerted a disproportionate influence on the overall pooled estimate. Nonetheless, readers should interpret the estimated prevalence rates with due caution, and these findings underscore the need for more comprehensive reporting of research in this field, irrespective of outcomes.

## Discussion

The flu vaccination rate among health care workers in China is only 27% well below the 75% target recommended by the WHO and behind the level in many high-income countries. However, our publication bias analysis (Egger's test, *P* = 0.008) suggests that this pooled estimate may be subject to overestimation due to potential underrepresentation of studies with null or negative findings. Consequently, the true coverage rate could be even lower, which reinforces the urgency of intervention. In the United States, for example, vaccination rates among health care workers have consistently exceeded 90%, largely owing to a comprehensive strategy that includes strict facility mandates and links to accreditation criteria (15). In contrast, vaccination policies for healthcare workers in China and many other Asian countries are largely voluntary and recommended in nature, which may in part explain the moderate uptake rates. Our finding that hospital requirements and free vaccination showed the strongest associations with uptake is consistent with international evidence suggesting that structural and policy-level interventions, in addition to awareness-raising activities, are critical to closing this gap. Our meta-analysis indicates that within a voluntary framework, institutional organization and the removal of economic barriers represent the most potent means, providing a clear roadmap for accelerating progress toward the WHO's objectives.

Subgroup analyses revealed notable variations in vaccination rates, yet the high residual heterogeneity (I^2^ > 90%) and non-significant meta-regression for most covariates (region, sample source, sampling type) indicate that these factors alone poorly explain between-study variance. We therefore supplemented our quantitative findings with a narrative synthesis to assess the consistency of direction and contextual plausibility of these patterns. For geographic distribution, vaccination rates descended from economically developed regions (30.20%) to moderately developed (25.40%) and underdeveloped ones (17.40%). Although meta-regression showed no statistical significance (*P* = 0.128), this gradient is narratively concordant with our core finding that free hospital-based vaccination (OR = 3.03) and higher monthly income (OR = 1.37) were robust correlates, suggesting that resource availability and affordability may contribute to regional disparities, even if study-level heterogeneity obscures a formal statistical interaction. In contrast, the unexpectedly higher rate in community settings (40.20%) vs. hospitals (19.17%) should be interpreted with caution: only six community studies were available, and all showed extreme heterogeneity, limiting the weight of this comparison in our narrative interpretation. The temporal increase was confirmed by meta-regression as a significant predictor (*P* = 0.006). Narratively, this steady upward trend aligns with the known cumulative effect of national policy promotion, growing institutional awareness, and the post-COVID-19 heightened perception of respiratory infection risk, which qualitatively supports a genuine improvement in coverage over time despite the substantial statistical heterogeneity. Overall, while pooled point estimates should be read cautiously given unexplained variance, the narrative consistency across multiple directional patterns reinforces the priority of structural policies (free access, institutional mandates) and time sensitive strategies.

### Policy and institutional factors are significantly associated with influenza vaccination among healthcare workers

Hospital-required vaccination (OR = 3.06, 95% CI = 2.63–3.56), free vaccination (OR = 3.03, 95% CI = 2.08–4.40) and free vaccination policy (OR = 3.20, 95% CI = 1.52–6.73) showed the strongest effects. This may be because the provision of vaccines at no cost directly removes the most important financial barrier, which is the basis for translating willingness to vaccate into practical action. The organization and requirement of the hospital level is to translate public health recommendations into an executable management process within the hospital, which greatly reduces the time and convenience costs of medical staff by setting up special vaccination sites in the hospital, coordinating resources and optimizing the process. These findings suggest that institutional organization and removal of economic barriers represent potentially impactful strategies within a voluntary framework. However, whether these observations justify a mandatory vaccination policy warrants caution. Our meta-analysis, being observational, cannot directly compare the effectiveness of mandates vs. voluntary approaches, nor does it capture cultural, ethical, or feasibility dimensions. We therefore suggest that Chinese policymakers explore mixed or risk-stratified approaches, for example, mandating vaccination in high-risk wards (ICUs, NICUs) while keeping it voluntary elsewhere, as a more nuanced, evidence-aligned strategy that balances patient protection with ethical feasibility and local acceptability. The South African experience highlights that even when willingness exists, vaccine unavailability can undermine uptake, reinforcing our conclusion that structural interventions are essential complements to educational campaigns (6). China's strategy should prioritize structural enablers while adapting successful educational and behavioral interventions from other LMIC settings.

Despite national guidelines recommending HCWs as a priority group, the implementation of free vaccination policies varies considerably across Chinese institutions and regions. This disparity stems from multiple factors. First, influenza vaccines in China are classified as non-National Immunization Program vaccines, meaning their provision and financing are largely determined at the local or institutional level rather than mandated nationally. Second, hospitals face competing resource priorities, and the cost of procuring vaccines for all staff can be substantial without dedicated government subsidies. Third, institutional leadership prioritization varies: some hospital administrators view HCW influenza vaccination as a patient safety and occupational health priority, while others do not.

### Prior vaccination history and vaccination intention were strongly associated with current influenza vaccination among healthcare workers

Meta-analysis of this study confirmed that previous vaccination history (OR = 2.73, 95% CI = 1.18–6.34, *P* = 0.019) and vaccination intention (OR = 6.66, 95% CI = 4.03–11.02, *P* < 0.001) were strongly associated with the current influenza vaccination among health care workers. This phenomenon can be interpreted from the perspective of behavioral science: previous successful vaccination experience and strong willingness can effectively reduce concerns about the safety of influenza vaccine, enhance the awareness of protection benefits, and reinforce vaccination as a habitual protective behavior. Thus increasing the influenza vaccination rate of health care workers. This finding has practical implications: it suggests that public health and hospital management strategies should focus on the first vaccination by facilitating interventions that enable health care workers to achieve the first vaccination, which is more likely to convert into stable protective habits.

### Knowledge of specific vaccination information is associated with influenza vaccination among healthcare workers

The meta-analysis of this study further explored the effect of knowledge cognition on vaccination behavior, and the results showed that among many knowledge factors, only the specific and actionable information of “knowing the vaccination time” had a clear statistical significance in promoting vaccination (OR = 2.81, 95% CI = 1.46–5.41, *P* = 0.002). In contrast, awareness of healthcare workers as a priority group, awareness of annual vaccination and belief of vaccine effectiveness did not show significant independent promoting effects. For this reason, general health literacy may not be sufficient to change behavior, and critical process-specific information - when and where to get the influenza vaccine - is the key pivot. In addition, there was heterogeneity in all cognitive factors (*I*^2^ > 88%), which may reflect the differences in organizational promotion and access to vaccination services in different hospitals. Future interventions should focus on providing clear, specific and timely vaccination guidelines, and combine these with convenient vaccination services to increase vaccination rates.

### Professional title is associated with influenza vaccination among healthcare workers

The results of this study showed that professional title showed a clear positive association for vaccination behavior, and the odds of vaccination increased with the increase of professional title (senior professional title OR = 1.35, *P* = 0.008). This may be due to the combination of greater occupational risk perception, role model responsibilities, and better access to organizational resources. In contrast, there was no significant difference in vaccination rates among different occupational types (doctors, nurses, and medical technicians), suggesting that health care workers in different positions may be more consistent in influenza exposure risk and health perception. Its high heterogeneity may indicate that the difference in vaccination is not caused by the occupation itself, but by the imbalance between different hospitals and departments.

### Demographic characteristics show modest associations with influenza vaccination among healthcare workers

The meta-analysis of this study further revealed the sociodemographic characteristics of vaccination behavior. Health care workers who were middle-aged (40–49 years old), female, and with higher monthly income (>3,000 yuan) were more likely to receive influenza vaccination. This pattern may reflect the influence of multiple factors: middle-aged medical workers are often in the critical period of their careers, and they have stronger awareness of health protection and professional responsibility; Women generally focus more on preventive care; And higher income may mean a greater willingness and ability to invest in health. More targeted publicity and convenience measures should be designed for young, male and low-income groups to reduce the gap in vaccination rates and improve the overall efficiency of intervention.

#### Certainty of evidence

We assessed the certainty of evidence for the primary outcome (pooled vaccination rate) and key associated factors using the GRADE framework. Observational cross-sectional studies start as low certainty. For the pooled vaccination rate, we downgraded one level for inconsistency (I^2^ = 99.91%, unexplained heterogeneity) and one level for publication bias (Egger's test, *P* = 0.008), resulting in very low certainty evidence. For the strongest facilitator, vaccination intention (OR = 6.66), we downgraded for inconsistency and imprecision (wide CI), but upgraded one level for strength of association (large effect size), yielding low certainty evidence. For structural factors (free vaccination, hospital requirements), evidence was rated low certainty due to observational design and inconsistency, though effect estimates were consistent in direction across studies. These ratings suggest that while pooled estimates provide useful directional signals, they should be interpreted with caution; high-quality prospective studies are needed to confirm causal relationships.

## Conclusions

The influenza vaccination rate among healthcare workers in China was 27% (95% CI = 21%−34%). This rate is relatively low and may compromise the effectiveness of influenza prevention and control. In this study, factors significantly associated with higher influenza vaccination rates included age 40–49 years, female sex, monthly income > 3,000 RMB, professional title, vaccination history, hospital attitude toward vaccination, free vaccination and policy, knowledge of vaccination schedule, and vaccination intention.

In summary, we therefore recommend that policymakers prioritize structural enablers, free on-site vaccination, institutional encouragement, and removal of access barriers, as immediate, evidence-supported interventions. Concurrently, pilot studies of mixed or risk-stratified approaches in high-risk departments should be conducted to assess feasibility, cost-effectiveness, and HCW acceptability before any consideration of broader mandatory policies. Such a stepped approach would generate the local evidence currently lacking and allow for culturally and ethically sensitive policy design. At the same time, in the medium to long term, complementary strategies should be developed to improve vaccination adherence, healthcare workers' attitudes toward vaccines should be reassessed, and there is also the potential to gradually move to non-mandatory approaches if deemed appropriate. thereby helping to protect vulnerable populations and reduce nosocomial infection risk.

### Limitations

This study has several limitations that should be considered when interpreting the findings. First, all studies were cross-sectional in design, which precludes the establishment of causal relationships between identified factors and vaccination behavior. Second, significant heterogeneity was observed across studies, which may reflect variations in regional policies, healthcare settings, and measurement methods, despite our use of random-effects models and subgroup analyses. Third, the review was limited to studies published in Chinese and English, possibly omitting relevant data. Fourth the generalizability of findings may be constrained by the uneven geographical distribution of included studies, with underrepresentation from certain regions of China.

## Data Availability

The original contributions presented in the study are included in the article/Supplementary material, further inquiries can be directed to the corresponding author.
