# Correction: Prevalence and factors influencing influenza vaccination among health care workers in China: a systematic review and meta-analysis

**DOI:** 10.3389/fpubh.2026.2007049

**Published:** 2026-10-05

**Authors:** Lina Xiong, Chunfang Cheng, Jie Yun

**Affiliations:** 1 College of Nursing, Chengdu University of Traditional Chinese Medicine, Chengdu, China; 2 Dazhou Central Hospital, Dazhou, Sichuan, China; 3 Hospital of Chengdu University of Traditional Chinese Medicine, Chengdu, China

**Keywords:** healthcare workers, influencing factors, influenza vaccination, meta-analysis, systematic review, vaccination coverage

Affiliation “College of Nursing, Chengdu University of Traditional Chinese Medicine, Chengdu, China” was omitted from the paper. This affiliation has now been added for author Lina Xiong.

Affiliation “Dazhou Central Hospital, Dazhou, Sichuan, China” was omitted from the paper. This affiliation has now been added for author Chunfang Cheng.

Affiliation “Hospital of Chengdu University of Traditional Chinese Medicine, Chengdu, China” was omitted from the paper. This affiliation has now been added for author Jie Yun.

Affiliation “Chengdu University of Traditional Chinese Medicine, Chengdu, China” was erroneously included in the paper. This affiliation has now been removed for authors Lina Xiong, Chunfang Cheng and Jie Yun.

The original version of this article has been updated.

