# Supplementary material for: Prevalence and factors influencing influenza vaccination among health care workers in China: a systematic review and meta-analysis
Source: Front Public Health. 2026 Sep 21;14:1831275. doi: 10.3389/fpubh.2026.1831275 (PMC13635197; doi:10.3389/fpubh.2026.1831275)
Supplement: Supplementary file 1 [file Data_Sheet_1.docx]

CNKI: (Subject: Medical Staff) OR (Subject: Healthcare Personnel) OR (Subject: Medical Technicians) OR (Subject: Paramedical Staff) OR (Subject: Doctors) AND (Subject: Influenza Vaccine) OR (Subject: Flu Virus Vaccine) OR (Subject: Influenza Virus Vaccine) AND (Subject: Vaccination Rate)

VIP: (((((((Title or Keywords=Medical Staff OR Title or Keywords=Healthcare Personnel) OR Title or Keywords=Nurses) OR Title or Keywords=Nursing) OR Title or Keywords=Doctors) OR Title or Keywords=Medical Technicians) AND ((Title or Keywords=Influenza Vaccine OR Title or Keywords=Influenza Virus Vaccine) OR Title or Keywords=Flu Virus Vaccine)) AND ((Title or Keywords=Vaccination Rate OR Title or Keywords=Influencing Factors) OR Title or Keywords=Related Factors))

WanFang: Subject:(Medical Staff or Healthcare Personnel or Nurses or Nursing or Doctors or Medical Technicians) and Subject:(Influenza Vaccine or Influenza Virus Vaccine or Flu Virus Vaccine) and Subject:(Vaccination Rate or Influencing Factors or Related Factors)

Pubmed：

("Medical Staff"[MeSH Terms] OR ("medical staffs"[Title/Abstract] OR "staff medical"[Title/Abstract] OR "staffs medical"[Title/Abstract])) AND ("Influenza Vaccines"[MeSH Terms] OR ("influenza virus vaccines"[Title/Abstract] OR "flu vaccines"[Title/Abstract] OR "influenza virus vaccine"[Title/Abstract] OR "vaccine influenza virus"[Title/Abstract] OR "virus vaccine influenza"[Title/Abstract] OR "influenza vaccine"[Title/Abstract] OR "vaccine influenza"[Title/Abstract] OR (("orthomyxoviridae"[MeSH Terms] OR "orthomyxoviridae"[All Fields] OR "Influenzavirus"[All Fields]) AND "Vaccines"[Title/Abstract]) OR "influenzavirus vaccine"[Title/Abstract] OR (("vaccin"[Supplementary Concept] OR "vaccin"[All Fields] OR "Vaccination"[MeSH Terms] OR "Vaccination"[All Fields] OR "vaccinable"[All Fields] OR "vaccinal"[All Fields] OR "vaccinate"[All Fields] OR "vaccinated"[All Fields] OR "vaccinates"[All Fields] OR "vaccinating"[All Fields] OR "vaccinations"[All Fields] OR "vaccination s"[All Fields] OR "vaccinator"[All Fields] OR "vaccinators"[All Fields] OR "vaccine s"[All Fields] OR "vaccined"[All Fields] OR "Vaccines"[Supplementary Concept] OR "Vaccines"[All Fields] OR "Vaccine"[All Fields] OR "Vaccines"[MeSH Terms] OR "vaccins"[All Fields]) AND "Influenzavirus"[Title/Abstract]) OR "flu vaccine"[Title/Abstract] OR "vaccine flu"[Title/Abstract] OR "trivalent live attenuated influenza vaccine"[Title/Abstract] OR "laiv vaccine"[Title/Abstract] OR "vaccine laiv"[Title/Abstract] OR "universal flu vaccines"[Title/Abstract] OR "universal influenza vaccines"[Title/Abstract] OR "universal influenza vaccine"[Title/Abstract] OR (("influenza s"[All Fields] OR "influenza, human"[MeSH Terms] OR ("Influenza"[All Fields] AND "human"[All Fields]) OR "human influenza"[All Fields] OR "Influenza"[All Fields] OR "influenzas"[All Fields] OR "influenzae"[All Fields]) AND "vaccine universal"[Title/Abstract]) OR "universal flu vaccine"[Title/Abstract] OR (("influenza, human"[MeSH Terms] OR ("Influenza"[All Fields] AND "human"[All Fields]) OR "human influenza"[All Fields] OR "Flu"[All Fields]) AND "vaccine universal"[Title/Abstract]) OR "high dose trivalent influenza vaccine"[Title/Abstract] OR "high dose trivalent influenza vaccine"[Title/Abstract] OR "quadrivalent influenza vaccine"[Title/Abstract] OR "influenza vaccine quadrivalent"[Title/Abstract] OR (("vaccin"[Supplementary Concept] OR "vaccin"[All Fields] OR "Vaccination"[MeSH Terms] OR "Vaccination"[All Fields] OR "vaccinable"[All Fields] OR "vaccinal"[All Fields] OR "vaccinate"[All Fields] OR "vaccinated"[All Fields] OR "vaccinates"[All Fields] OR "vaccinating"[All Fields] OR "vaccinations"[All Fields] OR "vaccination s"[All Fields] OR "vaccinator"[All Fields] OR "vaccinators"[All Fields] OR "vaccine s"[All Fields] OR "vaccined"[All Fields] OR "Vaccines"[Supplementary Concept] OR "Vaccines"[All Fields] OR "Vaccine"[All Fields] OR "Vaccines"[MeSH Terms] OR "vaccins"[All Fields]) AND "quadrivalent influenza"[Title/Abstract]) OR "trivalent influenza vaccine"[Title/Abstract] OR "influenza vaccine trivalent"[Title/Abstract] OR (("vaccin"[Supplementary Concept] OR "vaccin"[All Fields] OR "Vaccination"[MeSH Terms] OR "Vaccination"[All Fields] OR "vaccinable"[All Fields] OR "vaccinal"[All Fields] OR "vaccinate"[All Fields] OR "vaccinated"[All Fields] OR "vaccinates"[All Fields] OR "vaccinating"[All Fields] OR "vaccinations"[All Fields] OR "vaccination s"[All Fields] OR "vaccinator"[All Fields] OR "vaccinators"[All Fields] OR "vaccine s"[All Fields] OR "vaccined"[All Fields] OR "Vaccines"[Supplementary Concept] OR "Vaccines"[All Fields] OR "Vaccine"[All Fields] OR "Vaccines"[MeSH Terms] OR "vaccins"[All Fields]) AND "trivalent influenza"[Title/Abstract]) OR "intranasal live attenuated influenza vaccine"[Title/Abstract] OR "intranasal live attenuated influenza vaccine"[Title/Abstract] OR "monovalent influenza vaccines"[Title/Abstract] OR "monovalent influenza vaccine"[Title/Abstract] OR (("influenza s"[All Fields] OR "influenza, human"[MeSH Terms] OR ("Influenza"[All Fields] AND "human"[All Fields]) OR "human influenza"[All Fields] OR "Influenza"[All Fields] OR "influenzas"[All Fields] OR "influenzae"[All Fields]) AND "vaccine monovalent"[Title/Abstract]) OR (("vaccin"[Supplementary Concept] OR "vaccin"[All Fields] OR "Vaccination"[MeSH Terms] OR "Vaccination"[All Fields] OR "vaccinable"[All Fields] OR "vaccinal"[All Fields] OR "vaccinate"[All Fields] OR "vaccinated"[All Fields] OR "vaccinates"[All Fields] OR "vaccinating"[All Fields] OR "vaccinations"[All Fields] OR "vaccination s"[All Fields] OR "vaccinator"[All Fields] OR "vaccinators"[All Fields] OR "vaccine s"[All Fields] OR "vaccined"[All Fields] OR "Vaccines"[Supplementary Concept] OR "Vaccines"[All Fields] OR "Vaccine"[All Fields] OR "Vaccines"[MeSH Terms] OR "vaccins"[All Fields]) AND "monovalent influenza"[Title/Abstract]))) AND ("Vaccination Coverage"[MeSH Terms] OR ((("Coverage"[All Fields] OR "Coverages"[All Fields]) AND "Vaccination"[Title/Abstract]) OR "coverage vaccination"[Title/Abstract] OR "vaccination coverages"[Title/Abstract] OR "immunization coverage"[Title/Abstract] OR "coverage immunization"[Title/Abstract] OR (("Coverage"[All Fields] OR "Coverages"[All Fields]) AND "Immunization"[Title/Abstract]) OR "immunization coverages"[Title/Abstract]))

Embase

#1 'medical staff'/exp

#2 'staff, medical':ab,ti OR 'staffs, medical':ab,ti OR 'medical staffs':ab,ti OR 'medical workers':ab,ti OR 'healthcare workers':ab,ti OR 'hospital medical staff':ab,ti OR 'hospitalists':ab,ti OR 'medical hospital staff':ab,ti

#3 #1 or #2

#4 'influenza vaccine'/exp

#5 'flu vaccines':ab,ti OR 'flu vaccine':ab,ti OR 'vaccine, flu':ab,ti OR 'influenza vaccine':ab,ti OR 'vaccine, influenza':ab,ti OR 'influenzavirus vaccines':ab,ti OR 'influenzavirus vaccine':ab,ti OR 'vaccine, influenzavirus':ab,ti OR 'influenza virus vaccines':ab,ti OR 'influenza virus vaccine':ab,ti OR 'vaccine, influenza virus':ab,ti OR 'virus vaccine, influenza':ab,ti OR 'influenza vaccination':ab,ti

#6 #4 or #5

#7 'vaccination coverage'/exp

#8 'coverage, vaccination':ab,ti OR 'coverages, vaccination':ab,ti OR 'vaccination coverages':ab,ti OR 'immunization coverage':ab,ti OR 'coverage, immunization':ab,ti OR 'coverages, immunization':ab,ti OR 'immunization coverages':ab,ti OR 'coverage rate':ab,ti OR 'vaccination rate':ab,ti OR 'influencing factor':ab,ti OR 'associated factor':ab,ti

#9 #7 or #8

#10 'china':ab,ti OR 'chinese':ab,ti

#11 #3 AND #6 AND #9 AND #10

Web of Science

TS=(medical staff or Staff, Medical or Staffs, Medical or Medical Staffs or Medical workers or Healthcare workers or hospital medical staff or Hospitalists or medical hospital staff)

TS=(Influenza Vaccines or Flu Vaccines or Flu Vaccine or Vaccine, Flu or Influenza Vaccine or Vaccine, Influenza or Influenzavirus Vaccines or Influenzavirus Vaccine or Vaccine, Influenzavirus or Influenza Virus Vaccines or Influenza Virus Vaccine or Vaccine, Influenza Virus or Virus Vaccine, Influenza or Influenza Vaccination)

TS=（Vaccination Coverage or Coverage, Vaccination or Coverages, Vaccination or Vaccination Coverages or Immunization Coverage or Coverage, Immunization or Coverages, Immunization or Immunization Coverages or Coverage rate	or Vaccination rate	or Influencing factor or Associated factor）

TS=（China or Chinese）

Scopus

"medical staff" or "Staff, Medical" or "Staffs, Medical" or "Medical Staffs" or "Medical workers" or "Healthcare workers" or "hospital medical staff" or "Hospitalists" or "medical hospital staff"

"Influenza Vaccines" or "Flu Vaccines" or "Flu Vaccine" or "Vaccine, Flu" or "Influenza Vaccine" or "Vaccine, Influenza" or "Influenzavirus Vaccines" or "Influenzavirus Vaccine" or "Vaccine, Influenzavirus" or "Influenza Virus Vaccines" or "Influenza Virus Vaccine" or "Vaccine, Influenza Virus" or "Virus Vaccine, Influenza" or "Influenza Vaccination"

"Vaccination Coverage" or "Coverage, Vaccination" or "Coverages, Vaccination" or "Vaccination Coverages" or "Immunization Coverage" or "Coverage, Immunization" or "Coverages, Immunization" or "Immunization Coverages" or "Coverage rate " or "Vaccination rate " or "Influencing factor" or "Associated factor"

"China" or "Chinese"

Cochrane Library

#1 medical staff

#2 (Staff, Medical):ab,ti,kw or (Staffs, Medical):ab,ti,kw or (Medical Staffs):ab,ti,kw or (Medical workers):ab,ti,kw or (Healthcare workers):ab,ti,kw or (hospital medical staff):ab,ti,kw or (Hospitalists):ab,ti,kw or (medical hospital staff):ab,ti,kw

#3 #1 or #2

#4 Influenza Vaccines

#5 (Flu Vaccines):ab,ti,kw or (Flu Vaccine):ab,ti,kw or (Vaccine, Flu):ab,ti,kw or (Influenza Vaccine):ab,ti,kw or (Vaccine, Influenza):ab,ti,kw or (Influenzavirus Vaccines):ab,ti,kw or (Influenzavirus Vaccine):ab,ti,kw or (Vaccine, Influenzavirus):ab,ti,kw or (Influenza Virus Vaccines):ab,ti,kw or (Influenza Virus Vaccine):ab,ti,kw or (Vaccine, Influenza Virus):ab,ti,kw or (Virus Vaccine, Influenza):ab,ti,kw or (Influenza Vaccination):ab,ti,kw

#6 #4 or #5

#7 Vaccination Coverage

#8 (Coverage, Vaccination):ab,ti,kw or (Coverages, Vaccination):ab,ti,kw or (Vaccination Coverages):ab,ti,kw or (Immunization Coverage):ab,ti,kw or (Coverage, Immunization):ab,ti,kw or (Coverages, Immunization):ab,ti,kw or (Immunization Coverages):ab,ti,kw or (Coverage rate ):ab,ti,kw or (Vaccination rate ):ab,ti,kw or (Influencing factor):ab,ti,kw or (Associated factor):ab,ti,kw

#9 #7 or #8

#10 (China):ab,ti,kw or (Chinese):ab,ti,kw

#11 #3 and #6 and #9 and #10
